# *Burkholderia pseudomallei* pathogenesis in human skin fibroblasts: A Bsa type III secretion system is involved in the invasion, multinucleated giant cell formation, and cellular damage

**DOI:** 10.1371/journal.pone.0261961

**Published:** 2022-02-03

**Authors:** Anek Kaewpan, Taksaon Duangurai, Amporn Rungruengkitkun, Watcharamat Muangkaew, Tapanee Kanjanapruthipong, Niramol Jitprasutwit, Sumate Ampawong, Passanesh Sukphopetch, Narisara Chantratita, Pornpan Pumirat

**Affiliations:** 1 Department of Microbiology and Immunology, Faculty of Tropical Medicine, Mahidol University, Bangkok, Thailand; 2 Department of Companion Animal Clinical Sciences, Faculty of Veterinary Medicine, Kasetsart University, Bangkok, Thailand; 3 Department of Tropical Pathology, Faculty of Tropical Medicine, Mahidol University, Bangkok, Thailand; 4 Department of Immunology, Faculty of Medicine Siriraj Hospital, Mahidol University, Bangkok, Thailand; 5 Mahidol-Oxford Tropical Medicine Research Unit, Faculty of Tropical Medicine, Mahidol University, Bangkok, Thailand; East Carolina University Brody School of Medicine, UNITED STATES

## Abstract

*Burkholderia pseudomallei—*a causative agent of melioidosis that is endemic in Southeast Asia and Northern Australia—is a Gram-negative bacterium transmitted to humans via inhalation, inoculation through skin abrasions, and ingestion. Melioidosis causes a range of clinical presentations including skin infection, pneumonia, and septicemia. Despite skin infection being one of the clinical symptoms of melioidosis, the pathogenesis of *B. pseudomallei* in skin fibroblasts has not yet been elucidated. In this study, we investigated *B. pseudomallei* pathogenesis in the HFF-1 human skin fibroblasts. On the basis of co-culture assays between different *B. pseudomallei* clinical strains and the HFF-1 human skin fibroblasts, we found that all *B. pseudomallei* strains have the ability to mediate invasion, intracellular replication, and multinucleated giant cell (MNGC) formation. Furthermore, all strains showed a significant increase in cytotoxicity in human fibroblasts, which coincides with the augmented expression of matrix metalloproteinase-2. Using *B. pseudomallei* mutants, we showed that the *B. pseudomallei* Bsa type III secretion system (T3SS) contributes to skin fibroblast pathogenesis, but O-polysaccharide, capsular polysaccharide, and short-chain dehydrogenase metabolism do not play a role in this process. Taken together, our findings reveal a probable connection for the Bsa T3SS in *B. pseudomallei* infection of skin fibroblasts, and this may be linked to the pathogenesis of cutaneous melioidosis.

## Introduction

*Burkholderia pseudomallei* is a pathogenic Gram-negative bacterium that causes fatal melioidosis, a disease that is endemic in Southeast Asia and northern Australia. This bacterium is categorized by the U.S. Centers for Disease Control and Prevention (CDC) as a tier 1 threat that could be used as a biological weapon [1]. Over the past decade, the incidence of melioidosis in northern Australia during the rainy season was reported to be approximately 50 cases per 100,000 of the population [2]. A high fatality rate of up to 40% is associated with cases of septicemia, and acute pneumonia constitutes around 62% of worldwide melioidosis cases [3, 4]. Cases of septic shock resulting from septicemia were reported to be the major cause of death (around 90%), despite patients being treated [5]. Melioidosis remains a public health problem worldwide and currently, no effective vaccine or inhibitor to combat this disease is available.

*B. pseudomallei* can infect both humans and animals [6, 7]. Inhalation, cutaneous infection via inoculation or skin abrasion, and ingestion are the major routes of acquiring *B. pseudomallei*. Once *B. pseudomallei* enters the host body, it can invade and proliferate inside both phagocytic and non-phagocytic cells [6, 8]. Many studies have focused on the pathogenesis in respiratory epithelial and phagocytic cells that are only related to respiratory infection and the host immune response, respectively [8–10]. However, the study on pathogenesis of *B. pseudomallei* in skin infection is very limited.

Skin (cutaneous) infections are responsible for 13%–24% of clinical presentations of melioidosis cases [11], and these can be acute or chronic localized infections, presenting as ulcers, nodules, or skin abscesses. Other manifestations may include symptoms of fever and muscle aches. Cutaneous melioidosis can be classified as primary or secondary melioidosis. The primary skin melioidosis is commonly localized, however; it has been described to be related with necrotizing fasciitis, internal organ abscesses, and sepsis [12]. It has been noted that some chronic or asymptomatic cases of skin melioidosis develop into secondary melioidosis, which can be fatal without appropriate treatment [12]. To date, the underlying mechanism of how *B. pseudomallei* cause the skin melioidosis remains poorly understood.

Invasion, intracellular survival, cell—cell spreading, and host cell damage are crucial for disease progression of *B. pseudomallei* infection. This process requires bacterial virulence factors. Several previous studies reported that the Bsa type III secretion system (T3SS), O-polysaccharide (OPS), capsular polysaccharide (CPS), and metabolic enzyme short-chain dehydrogenase (SDO) are important virulence factors for establishing a successful infection of *B. pseudomallei* in both phagocytic and non-phagocytic cells [7, 13]. For example, BsaQ (*bpss1543*) is a structural component of the Bsa T3SS. Muangsombut et al. [14] demonstrated that *B. pseudomallei bsaQ* mutation resulted in decreased efficiencies of plaque formation of HeLa human epithelial cells, invasion into A549 human respiratory epithelial cells, and multinucleated giant cell (MNGC) formation in J774A.1 murine macrophage cells. Similarly, BipB (*bpss1532*) is a needle component of Bsa T3SS. Suparak et al. [15] showed that genetic inactivation of *B. pseudomallei bipB* reduced cell-to-cell spreading of bacteria in HeLa cells. The *bipB* mutant also exhibited decreased MNGC formation and induction of apoptosis of J774A.1 macrophages [15]. Additionally, ChbP (*bpss1385*) is an effector of the Bsa T3SS. A previous study reported that *B. pseudomallei chbP* mutant caused reduction of plaque formation in HeLa cells, as well as declined levels of lactate dehydrogenase (LDH; indicator of cytotoxicity) in HeLa cells and U937 human macrophage cells [16]. It has also been reported that OPS is associated with antigenic variation and internalization of *B. pseudomallei* into human monocytic THP-1 cell line, whereas CPS enables bacterial spreading to other sites of infection [17]. Recently, *B. pseudomallei* SDO encoded by *bpss2242* gene was identified [18]. This SDO is a metabolic enzyme harboring glucose dehydrogenase activity that catalyzes the following chemical reaction: D-glucose + NAD^+^  =  D-glucono-1,5-lactone + NADH + H^+^. Inactivation of SDO (*bpss2242*) affects *B. pseudomallei* adherence to A549 human respiratory and HFF-1 human skin fibroblast cell lines [18], invasion into A549 cells, and early survival in J774.1 cells [19]. As mentioned above, it might indicate a common pathogenesis among phagocytic and non-phagocytic host cells that is mediated by these *B. pseudomallei* virulence factors. Interestingly, one study showed that there is a correlation between the absence of a filamentous hemagglutinin gene (*fhaB3*; encoded by *bpss2053*) and localized skin lesions without sepsis [20]. Whereas, the presence of *fhaB3* is associated with positive blood culture [20]. Even though *fhaB3+* and *fhaB3-* clinical isolates exist naturally in the population of *B. pseudomallei*, lack of *fhaB3* gene may be involved in the pathogenesis of *B. pseudomallei* in skin infection.

In the present study, we focused on the investigation of *B. pseudomallei* pathogenesis in skin fibroblast cells, which is a resident cellular component of the dermis [21]. Fibroblasts are responsible for production of extracellular matrix proteins that are critical in maintaining the skin homeostasis and are involved the wound healing process. Fibroblasts are among the most used cell models for studying microbial pathogenesis. The roles of fibroblasts in skin infection have been well established in several common skin pathogens, such as Staphylococcus aureus [22], Candida albicans [23], and Varicella-Zoster Virus [24]. Despite extensive studies in other pathogens, human skin fibroblasts have not previously been investigated in *B. pseudomallei* infection. There is no direct evidence of skin fibroblast infection in individuals with cutaneous melioidosis. The link between skin fibroblast and cutaneous melioidosis arises from the most common dermal presentation, being an ulcer (with or without a purulent exudate) [12]. We hypothesized that *B. pseudomallei* is able to infect the human skin fibroblasts, and its virulence factors facilitate this process. Therefore, we studied *B. pseudomallei* pathogenesis using the human skin fibroblast HFF-1 cell line, and we assessed invasion, intracellular replication, MNGC formation, and host cell damage. Additionally, we examined the virulence factors that contribute to the invasion, intracellular survival, and cytotoxicity of *B. pseudomallei* in human skin fibroblasts. *B. pseudomallei* mutants that were defective in each of these components, that is, Bsa T3SS (*bsaQ*, *bipB*, *chbP*), OPS (*oacA*, *wbiD*, *wbiA*), CPS (*wcbB*), and SDO metabolism (*sdo*), were analyzed to determine their phenotypes in human skin fibroblasts. Additionally, *B. pseudomallei fhaB3*+ (A16 and A19) and *fhaB3*- (A8 and A24) isolates were included in this study to test the importance of FhaB3 in *B. pseudomallei* pathogenesis in HFF-1 human skin fibroblasts.

## Materials and methods

### Ethics statement

All experiments and methods were performed in accordance with relevant guidelines and regulations. This project has been approved from the ethics committee of Faculty of Tropical Medicine, Mahidol University, Bangkok, Thailand (Reference No: TMEC 20–057).

### Biosecurity aspects

General bacterial laboratory facilities were operated following all the security and safety regulations of our university. This is a BSL2 enhance facility that is currently being upgraded to BSL3 practices.

This project has been approved from the biosafety committee of Faculty of Tropical Medicine, Mahidol University, Bangkok, Thailand (Reference No: FTM-IBC-18-02).

### Bacterial strains, cell line and growth conditions

Five wild-type and nine mutant strains of *B. pseudomallei* were obtained from previous studies (Table 1). All bacterial strains were cultured in lysogeny broth (LB) (BD) at 37°C with shaking and supplemented as required with antibiotics.

**Table 1:** Bacterial wild-type and mutant strains used in this study.

Bacterial strain	Description	Source or reference
**Reference clinical strain**		
**K96243**	Wild-type	[45]
**Clinical strain**		
**A8**	Wild-type, clinical strain, *FhaB3* negative	[20]
**A16**	Wild-type, clinical strain, *FhaB3* positive	[20]
**A19**	Wild-type, clinical strain, *FhaB3* positive	[20]
**A24**	Wild-type, clinical strain, *FhaB3* negative	[20]
**Bsa T3SS mutant**		
***bipB***	K96243 derivative: Δ*bipB* (Mutant defective in Bsa T3SS translocator)	[15]
***bipB’***	K96243 derivative with Δ*bipB* complemented with *bipB*	This study
***bsaQ***	K96243 derivative: Δ*bsaQ* (Mutant defective in Bsa T3SS apparatus)	[14]
***bsaQ’***	K96243 derivative with Δ*bsaQ* complemented with *bsaQ*	This study
***chbP***	K96243 derivative: Δ*chbP* (Mutant defective in Bsa T3SS effector CHBP)	[16]
***chbP’***	K96243 derivative with Δ*chbP* complemented with *chbP*	[16]
**OPS mutant**		
***oacA***	K96243 derivative: Δ*oacA* (Mutant defective in acetylation of OPS)	[17]
***wbiA***	K96243 derivative: Δ*wbiA* (Mutant defective in acetylation of OPS)	[17]
***wbiD***	K96243 derivative: Δ*wbiD* (Mutant defective in OPS synthesis)	[17]
**CPS mutant**		
***wcbB***	4095a derivative: Δ*wcbB* (Mutant defective in CPS)	[17]
**SDO metabolism mutant**		
***sdo***	K96243 derivative: Δ*sdo* (Mutant defective in Glucose dehydrogenase activity)	[19]

The human skin fibroblast HFF-1 cell line (ATCC® SCRC1041™, Manassas, VA) was cultured and maintained in RPMI 1640 medium (Gibco BRL) supplemented with 10% (v/v) heat-inactivated fetal bovine serum (Gibco BRL) and 1X Penicillin-Streptomycin combined antibiotic (Gibco BRL). The cells were incubated at 37°C in a humidity-controlled incubator with 5% Carbon dioxide (CO_2_) condition throughout the study. HFF-1 cells were harvested with 0.25% (v/v) trypsin-EDTA solution at approximately 90% confluency.

### Construction of complemented strains

The open-reading frame of *bipB* and *bsaQ* genes was amplified from *B. pseudomallei* K96243 genomic DNA using designed primers (S1 Table). The amplified DNA fragment of *bipB* (1,888 bp) and *bsaQ* (2,098 bp) were cloned into the broad-host-range cloning vector pBBR1MCS [25]. The constructed plasmids harbouring *bipB* and *bsaQ* were delivered into the *B. pseudomallei bipB* and *bsaQ* mutants by conjugation to give *B. pseudomallei bipB* and *bsaQ* complemented strains, respectively. The complementation was confirmed by plasmid DNA extraction, PCR, and sequencing.

### Growth kinetics of *B*. *pseudomallei*

An isolated colony of *B. pseudomallei* was inoculated in LB broth, and incubated at 37°C with shaking at 200 rpm for 24 h. Then, the overnight-culture of bacteria was washed with phosphate buffer saline (PBS), and adjusted to an optical density (OD_600_) of 0.5. To examine the growth kinetics, the adjusted *B. pseudomallei* was inoculated at a ratio of 1:500 into fresh LB medium, and incubated at 37°C with shaking at 200 rpm. At determined time points, OD_600_ measurement was performed.

### Invasion assay and intracellular replication assay

One day before infection, 5x10^4^ cells of HFF-1 human skin fibroblasts were seeded into each well of a 24-well plate. Bacteria from an overnight culture were adjusted to 1x10^6^ cells per ml, and added into PBS-washed human skin fibroblasts at the indicated multiplicity of infection (MOI). The infected cells were incubated at 37 ^๐^C in 5% CO_2_. At 2 h post-infection, the infected cells were washed with PBS twice before adding fresh RPMI 1640 medium containing 250 μg per ml of kanamycin (or 128 μg/mL gentamicin and 256 μg/mL amikacin) to eliminate extracellular bacteria. At 4 h post-infection, the infected human skin fibroblasts were washed with PBS and lysed with 0.1% (v/v) Triton X-100. A serial dilution was made, and 10 μl of each dilution was dropped onto LB agar plate and incubated for 24–48 h. A colony forming unit (CFU) was counted to estimate the number of invading bacteria. The percentage of invasion efficiency is calculated using the equation: (number of intracellular bacteria at 4 h post-infection/ number of bacteria added) x 100. For intracellular replication, the numbers of viable bacteria recovered at 6, 8, and 10 h post-infection were counted as CFU as described previously.

### Multinucleated giant cell (MNGC) formation assay

The HFF-1 Human skin fibroblasts were seeded on a cover slip and infected with *B. pseudomallei* strains at a MOI of 20:1. The number of MNGCs was determined at 6, 8, 10 h post-infection, as previously described [26, 27]. Briefly, the infected cells were washed thrice with PBS before fixing with 4% (v/v) of paraformaldehyde in PBS. The fixed cells were then soaked in 50% (v/v) and 90% (v/v) ethanol; respectively, and then rinsed twice with distilled water. Next, the fixed cells were stained using Giemsa stain (Sigma) for 5 min, and then rinsed in distilled water before mounting in 100% glycerol. Images were acquired on an Olympus BX41 microscope. MNGCs were considered as cells containing at least three nuclei. The percentage of MNGC formation was calculated using the following formula: (number of nuclei in a multinucleated giant cell/total number of nuclei counted) x 100. A minimum of 1,000 nuclei was counted for each experiment and time point.

The severity of MNGC formation was determined using H-score criteria as described below.


$$\begin{array}{cc} Category & Nuclei\;criteria\\ 1 & 3‐5\\ 2 & 6‐10\\ 3 & >7 \end{array}$$

MNGC H-score = (% MNGC at Category 1 x 1) + (% MNGC at Category 2 x 2) + (% MNGC at Category 3 x 3)

### Lactate dehydrogenase (LDH) detection

Lactate dehydrogenase (LDH) released from damage cells was assessed using the CytoTox96 kit (Promega, Madison, WI) according to the manufacturer’s instructions. The percentage of cytotoxicity was calculated by using the following equation: (OD_490_ experimental release—OD_490_ spontaneous release)/(OD_490_ maximum release—OD_490_ spontaneous release) x100. The amount of LDH released from uninfected cells was considered as a spontaneous release, whereas the maximum release of LDH was obtained by lysing of uninfected cells with 0.1% Triton X-100.

### MTT (methyl thiazolyl tetrazolium) assay

MTT assay on the cellular metabolic activity of human skin fibroblasts was performed according to the previous study [28] with some modifications. After 10 h post-infection using MOI 20:1 as described above, 10 μl of 0.1 mg/mL MTT solution was added to the infected-fibroblast cells in each well, and incubated for 5 h at 37°C. Then, the culture media was discarded and 100 μl of dimethyl sulfoxide (DMSO) was added to each well. The MTT plates were further incubated at 37°C for 10 min and measured at the absorbance 570 nm. The percentage of cellular metabolic activity was expressed as a percentage relative to an uninfected control.

### Detection of MMP-2 and MMP-9 expression

At 10 h post-infection, the total RNA from *B. pseudomallei*-infected cells was extracted using RNeasy Mini Kit (QIAGEN) and treated with DNase. Real time RT-PCR was performed according to the manufacturer’s instructions. The RT-derived cDNA was used as the template in the reaction containing the primers listed in S1 Table. RPL19 was used as the internal reference for normalization. The thermocycling conditions were as follows: 95°C for 2 min; 95°C for 30 sec, 60°C for 1 min for 30 cycles; 72°C for 5 min, on a CFX96 Touch™ Real-Time PCR Detection System (Bio-Rad, Singapore). Fold change in expression was calculated by 2^−ΔΔCT^ using the relative mRNA level of uninfected cells.

### Electron microscopic assay

The infected cells were collected using 0.25% (w/v) Trypsin-EDTA before adding pre-warmed RPMI and being harvested by centrifugation. Then, the cells were fixed in sucrose phosphate buffer (SPB, pH 7.4) containing 2.5% (v/v) glutaraldehyde at 25°C for 1 h. After that, the fixed cells were rinsed in SPB and post-fixed with 1% (w/v) osmium tetroxide (Electron Microscopy Sciences Inc.) for 1 h. Then, the cells were incubated in 0.5% (w/v) tannic acid (Merck) for 30 min. Dehydration was obtained using a graded series of ethanol solutions (from 30%, 50%, and 70% (v/v), respectively) before embedding the samples in LR White resin at 65°C for 48 h. Ultrathin sections were cut using a Reichert Ultracut microtome (Leica), and stained with uranyl acetate/lead acetate before examining under a H7700 transmission electron microscope (Hitachi, Japan).

### Statistical analysis

Each experiment was conducted in triplicate. Statistical analysis was performed using the GraphPad Prism 6 program (STATCON). Student *t*-test of three independent experiments was used to determine the difference in quantitative data among different groups. All data in this study were presented as mean ± standard deviation. The *p*-value ≤ 0.05 was considered as statistically significant.

## Results

### Invasion of human skin fibroblast cells by *B*. *pseudomallei*

In total, 13 different *B. pseudomallei* strains were included in this study to analyze pathogenicity during skin fibroblast infection. We first determined the growth of the *B. pseudomallei* strains in LB and RPMI media by OD_600_ measurements. In each medium, the growth kinetics of all *B. pseudomallei* strains were comparable. In LB, each bacterial strain was grown until the OD_600_ reached 0.5 at 8 h and then reached around 3.5 at 18 h after inoculation, whereas the OD_600_ reached only about 0.2 in RPMI medium after 24 h after inoculation. However, there was no significant difference among these bacterial strains either in LB or RPMI media (S1 Fig).

Next, the ability of *B. pseudomallei* to invade HFF-1 human skin fibroblast cells was examined by infection using a range of multiplicity of infection (MOI) values and the number of intracellular bacteria was determined at 4 h post-infection. *B. pseudomallei* K96243 was able to enter human skin fibroblast cells at a MOI of 20:1 with an invasion efficiency of approximately 0.1%, whereas no bacteria were recovered from the infected cells using a MOI of 10:1 or 1:1 (Table 2). The percentage of invasion was increased 10-fold when *B. pseudomallei* K96243 was cultured in human skin fibroblasts at a MOI of 100:1 or 200:1 (Table 2). However, we observed that the infected human skin fibroblast cell monolayer became rapidly detached when a MOI of 100:1 or 200:1 was used. Therefore, we selected a MOI of 20 for further investigations. The presence of intracellular *B. pseudomallei* in the HFF-1 skin fibroblast cells was verified by transmission electron microscopy and showed that *B. pseudomallei* was internalized within the host cells at 4 h post-infection (S2 Fig).

**Table 2:** Invasion of *B*. *pseudomallei* K96243 into the HFF-1 human skin fibroblasts.

MOI (Bacteria:Host)	Mean no. of invading bacteria (cfu/well)	Invasion Efficiency (%)
1:10	ND	ND
10:1	ND	ND
20:1	1.20 x10^3^ ± 0.34 x10^3^	0.12 ± 0.03
100:1	6.23 x10^4^ ± 1.79 x10^4^	1.20 ± 0.29
200:1	1.52 x10^5^ ± 0.90 x10^5^	1.19 ± 0.52

ND, Not detected.

**Fig 1:**
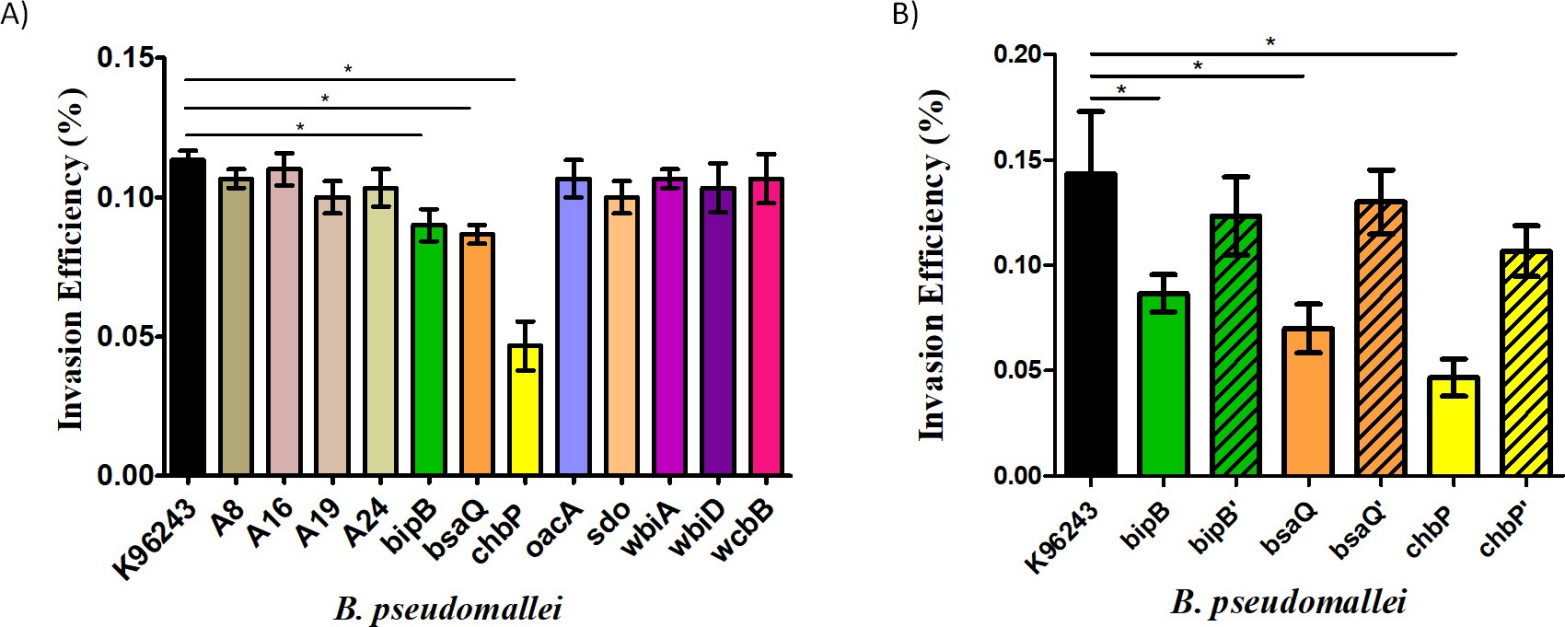
Invasion efficiency of *B*. *pseudomallei* into human skin fibroblasts. HFF-1 human skin fibroblast cells were infected with *B. pseudomallei* at a MOI of 20. The invading bacteria into the human skin fibroblasts were recovered at 4 h post-infection. A) *B. pseudomallei* reference strain K96243, two clinical isolates of *fhaB3*+ strains (A16 and A19), two clinical isolates of *fhaB3*- strains (A8 and A24), and eight mutant strains that comprise three mutants for which the Bsa T3SS is defective (*bipB*, *bsaQ*, *chbP*), three mutants for which OPS is defective (*oacA*, *wbiA*, *wbiD*), a mutant for which CPS is defective (wcbB), and a mutant for which SDO metabolism is defective (*sdo*). B) *B. pseudomallei* wild-type strain K96243, Bsa T3SS mutants (*bipB*, *bsaQ*, *chbP*), and complemented strains (*bipB*’, *bsaQ*’, *chbP*’) in the Bsa T3SS. Values represent the mean ± standard deviation from three independent experiments. * *p* ≤ 0.05, indicating a significant difference compared with reference strain K96243.

A previous study has shown that the absence of a filamentous hemagglutinin gene, *fhaB3*, correlated with localized skin lesions without sepsis [20], and therefore we hypothesized that *fhaB3* might affect the ability of *B. pseudomallei* to invade skin fibroblasts. *B. pseudomallei* isolates from melioidosis patients containing *fhaB3* (A16 and A19) or lacking *fhaB3* (A8, A24) were investigated. The results showed that the invasion efficiency of *B. pseudomallei* lacking *fhaB3* was comparable with that of *B. pseudomallei* harboring *fhaB3* and no significant difference was detected, compared with *B. pseudomallei* K96243 reference strain (p > 0.05) (Fig 1A). This implies that *fhaB3* is not associated with *B. pseudomallei* invasion of human skin fibroblast cells.

Other specific virulence factors of *B. pseudomallei* may be required for the invasion of HFF-1 human skin fibroblasts. To address the role of *B. pseudomallei* virulence factors in cellular invasion, eight mutants defective in the T3SS (*bipB*, *bsaQ*, and *chbP*), OPS (*oacA*, *wbiA*, *wbiD*), CPS (*wcbB*), and the metabolic enzyme SDO (*sdo*) were evaluated. Fig 1A shows that only three Bsa T3SS mutants were impaired in the invasion of HFF-1 skin fibroblasts, while the other mutant strains showed a similar level of invasion as the wild-type reference strain. Interestingly, the *chbP* mutant of *B. pseudomallei* showed the lowest percentage of skin fibroblast invasion (Fig 1A), and the *bipB* and *bsaQ* mutants of *B. pseudomallei* also showed defective invasion into skin fibroblast. The ability to invade skin fibroblast cells could be restored by complementation of the *bipB*, *bsaQ*, and *chbP* mutant strains (Fig 1B). These data suggest that the Bsa T3SS is involved in the invasion of *B. pseudomallei* into HFF-1 human skin fibroblast cells.

### Intracellular replication of *B*. *pseudomallei* in human skin fibroblasts

**Fig 2:**
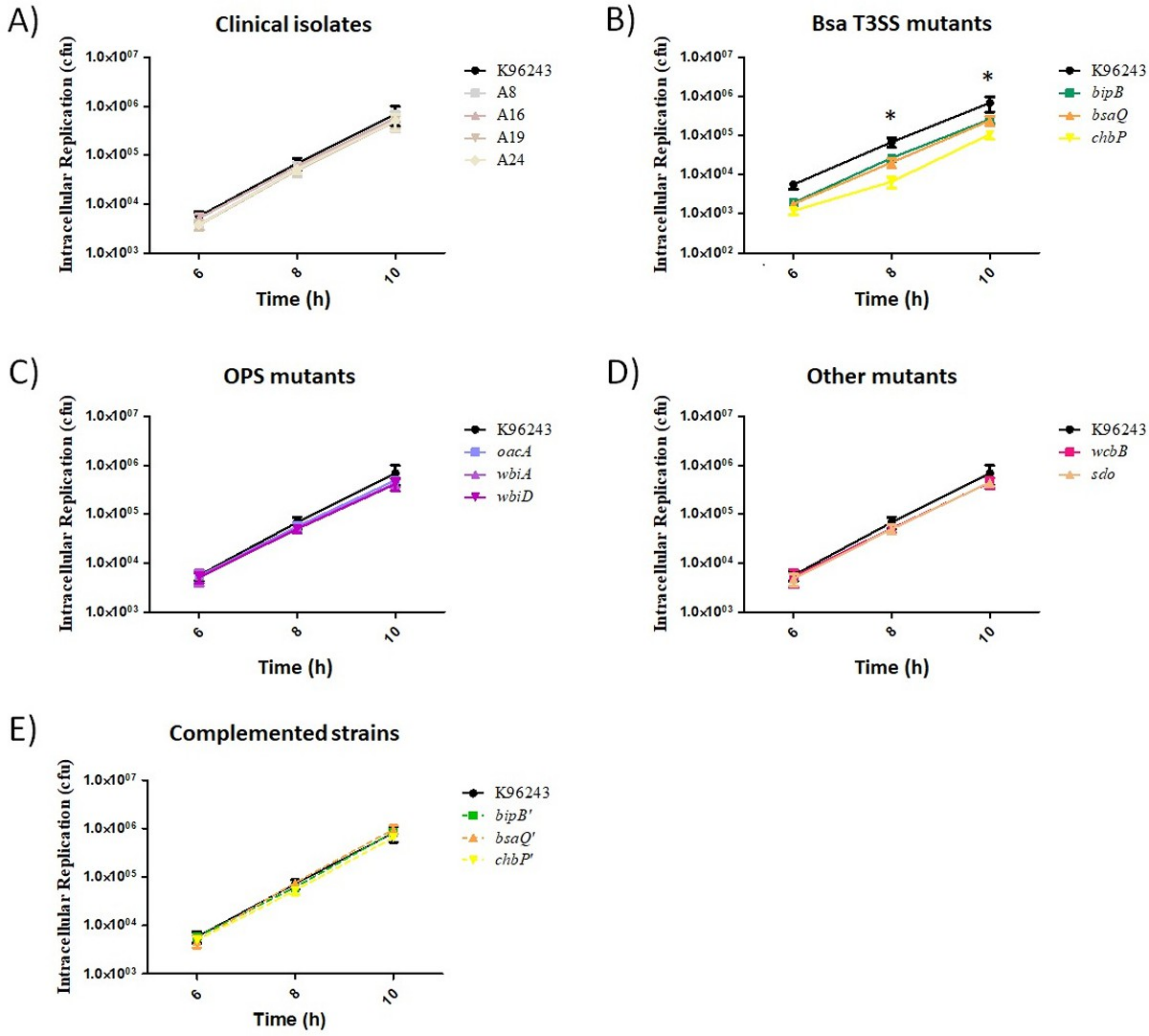
Intracellular survival of *B*. *pseudomallei* in human skin fibroblasts. HFF-1 human skin fibroblast cells were infected with different strains of *B. pseudomallei* at a MOI of 20. Intracellular multiplication was determined at 6, 8, and 10 h post-infection. A) *B. pseudomallei* reference strain K96243, two clinical isolates of *fhaB3*+ (A16 and A19), and two clinical isolates of *fhaB3*- (A8 and A24). B) *B. pseudomallei* mutants defective in the Bsa T3SS (*bipB*, *bsaQ*, *chbP*). C) *B. pseudomallei* mutants defective in OPS synthesis (*oacA*, *wbiA*, *wbiD*). D) *B. pseudomallei* mutants defective in the production of CPS (wcbB) or SDO metabolism (*sdo*). E) *B. pseudomallei* complemented strains (*bipB*’, *bsaQ*’, *chbP*’) in the Bsa T3SS. The same data set for reference strain K96243 was included in each panel. Values represent the mean ± standard deviation from three independent experiments. * *p* ≤ 0.05 indicates a significant difference compared with reference strain K96243.

Following invasion, intracellular bacteria can replicate and survive inside host cells. The capacity of *B. pseudomallei* to replicate in human skin fibroblasts has not been reported; although human gingival fibroblasts (HGFs) have been investigated previously [29]. Thus, we determined the intracellular replication and survival of *B. pseudomallei* by co-culture with HFF-1 human skin fibroblast cells using the optimized MOI of 20, as described above. The number of bacteria internalized into human fibroblasts was assessed every 2 h until 10 h post-infection. The results showed that all *B. pseudomallei* strains used in this study could replicate and survive inside human skin fibroblast cells (Fig 2). Similar to the invasion data, there was no significant difference in the number of recovered intracellular bacteria at each time point when comparing *B. pseudomallei fhaB3*+ and *fhaB3*- strains with the K96243 reference strain (Fig 2A).

Intracellular survival of *B. pseudomallei* in human skin fibroblasts was analyzed using the mutants lacking the defined virulence factor. Only Bsa T3SS mutant strains showed a lower number of viable bacteria recovered from the infected-human skin fibroblasts at 6, 8, and 10 h post-infection (Fig 2B), whereas the higher number of viable bacteria recovered from other *B. pseudomallei* mutants, including *oacA*, *wbiA*, *wbiD*, *wcbB*, and *sdo* mutants, was similar to that of the reference strain K96243 (Fig 2C and 2D). Meanwhile, the number of intracellular bacteria at 10 h was further compared with the bacterial number of the initial inoculum at 4 h post-infection. We found that multiplication of *B. pseudomallei* wild-type strains showed approximate 2-, 20-, and 200-fold increases at 6, 8, and 10 h, respectively, compared with 4 h post-infection (S2 Table). On the other hand, the bacterial numbers of the *bipB*, *bsaQ*, and *chbP* mutants at 10 h increased approximately 150-, 160-, and 90- fold compared with those at 4 h post-infection (S2 Table). These data indicated that all mutants are able to survive and replicate inside HFF-1 human skin fibroblasts. However, the low number of invading bacteria may affect the number of recovered intracellular bacteria at a later time point.

Accordingly, we suspected that a low number of invading bacteria may affect the replication rate of intracellular bacteria of the Bsa T3SS mutant within HFF-1 human skin fibroblasts. Thus, the doubling time of intracellular bacteria was further analyzed. We found that doubling time of *B. pseudomallei* Bsa T3SS mutants in HFF-1 skin fibroblast cells showed no significant difference from *B. pseudomallei* wild-type strains (S2 Table). We concluded that the inactivation of *bipB*, *bsaQ*, and *chbP* genes did not impair the intracellular replication of *B. pseudomallei* in HFF-1 human skin fibroblasts.

### *B*. *pseudomallei* can induce MNGC formation in human skin fibroblasts

**Fig 3:**
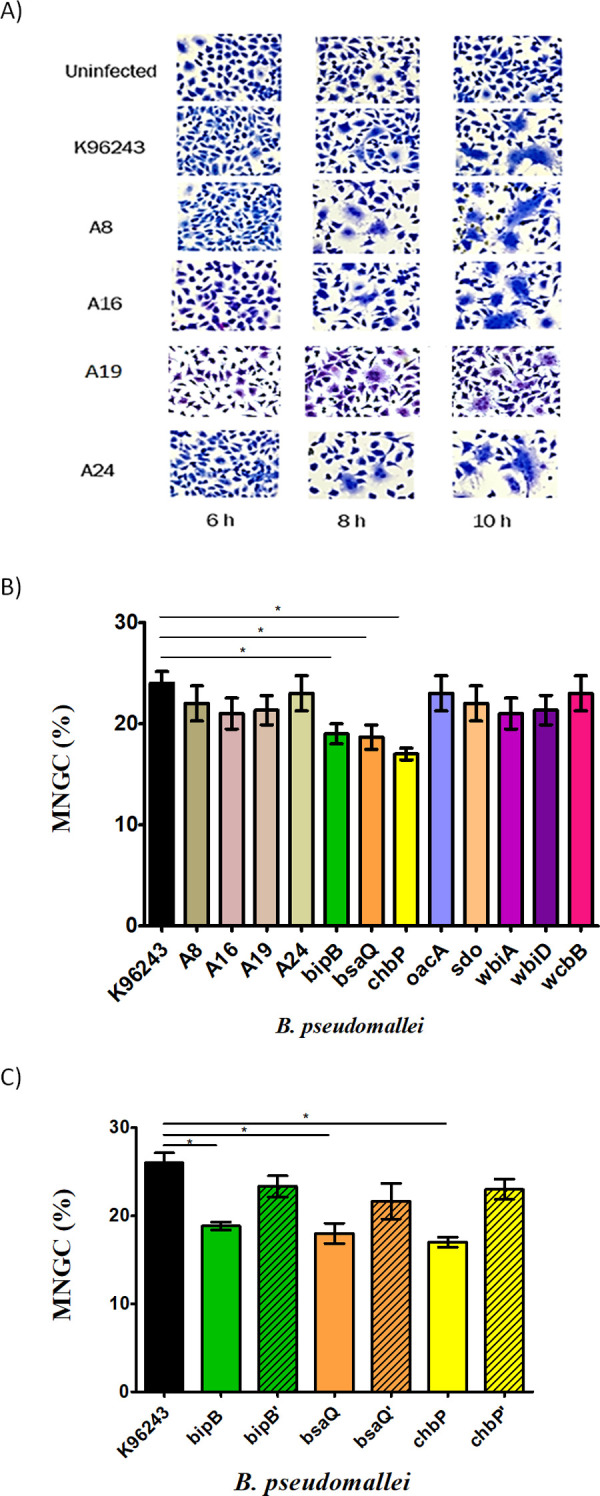
MNGC formation by *B*. *pseudomallei* in human skin fibroblasts. HFF-1 human skin fibroblasts were infected with a MOI of 20 for 10 h. Infected cells were stained with Giemsa’s solution. MNGC were counted under a 40× magnitude objective lens, for at least 1000 nuclei. A) Representative images of MNGC formation of HFF-1 human skin fibroblast cells induced by *B. pseudomallei* reference strain K96243, two clinical isolates of *fhaB3*+ (A16 and A19), and two clinical isolates of *fhaB3*- (A8 and A24). MNGC are defined as cells that contain at least three nuclei as well as intracellular bacteria. B) Percentage of MNGC formed by *B. pseudomallei* reference strain K96243, two clinical isolates of *fhaB3*+ strains (A16 and A19), two clinical isolates of *fhaB3*- strains (A8 and A24), and eight mutant strains that comprise three mutants for which the Bsa T3SS is defective (*bipB*, *bsaQ*, *chbP*), three mutants for which OPS is defective (*oacA*, *wbiA*, *wbiD*), a mutant for which CPS is defective (wcbB), and a mutant for which SDO metabolism is defective (*sdo*). C) Percentage of MNGC formed by *B. pseudomallei* wild-type K96243, Bsa T3SS mutants (*bipB*, *bsaQ*, *chbP*), and complemented strains (*bipB*’, *bsaQ*’, *chbP*’). The percentage of MNGC is the number of MNGC divided by 1000, then multiplied by 100. Values represent the mean ± standard deviation from three independent experiments. * *p* ≤ 0.05 indicates a significant difference compared with reference strain K96243.

*B. pseudomallei* has been shown to spread into nearby host cells and induce those cells to form MNGC [6]. The formation of MNGC assists *B. pseudomallei* to survive under potentially harmful conditions, such as in the presence of antimicrobial agents or antibodies [30]. This characteristic appears to be related to pathogenicity of *B. pseudomallei* [31]. In this study, we investigated MNGC formation in HFF-1 human skin fibroblast cells. *B. pseudomallei* K96243, and *fhaB3*+ and *fhaB3*- isolates, induced human skin fibroblast cells to form MNGC at 8 h post-infection (Fig 3A). By 10 h post-infection, no significant difference was detected in the number of MNGC among these strains (Fig 3B).

HFF-1 skin fibroblasts infected by eight different *B. pseudomallei* mutants were monitored for MNGC formation (Fig 3B). Interestingly, we found that the induction of MNGC formation by the *bipB*, *bsaQ*, and *chbP* mutants was significantly decreased compared with reference strain K96243 at 10 h post-infection.

The degree of MNGC formation of HFF-1 human skin fibroblasts induced by *B. pseudomallei* strains at 10 h was further evaluated using the H-score, which is a quantitative measure of the degree of MNGC formation in the range of 0 to 300. The results showed that the H-score of reference strain K96243 and four cutaneous isolates were comparable, ranging from 110 ± 0.6 to 115 ± 2.9 (Table 3). *B. pseudomallei* mutants, including OPS, CPS and SDO-defective mutants, showed similar level of H-score of the wild-type strains. However, the H-scores of the *bipB*, *bsaQ*, and *chbP* mutants were significantly less than that of reference strain K96243 (Table 3), approximately 104 ± 1.2, 104 ± 1.7, and 101 ± 2.3, respectively. This indicated that the Bsa T3SS plays a role in inducing MNGC formation in human skin fibroblast by *B. pseudomallei*.

**Table 3:** MNGC severity H-score of HFF-1 human skin fibroblasts infected by *B*. *pseudomallei* skin clinical and reference strains.

*B*. *pseudomallei* strain	Severity	*p-*value
K96243	115±2.9	-
A8	112±1.2	0.3892
A16	114±1.7	0.7812
A19	113±1.2	0.5551
A24	110±0.6	0.1647
*bipB*	104±1.2	0.0241*
*bsaQ*	104±1.7	0.0309*
*chbP*	101±2.3	0.0193*
*oacA*	113±2.6	0.8110
*sdo*	112±2.9	0.5032
*wbiA*	111±2.3	0.3401
wbiD	114±1.2	0.7638
*wcbB*	110±1.7	0.2117

* *p* ≤ 0.05, indicating a significant difference compared with reference strain K96243.

### *B*. *pseudomallei* causes damage of human skin fibroblasts

**Fig 4:**
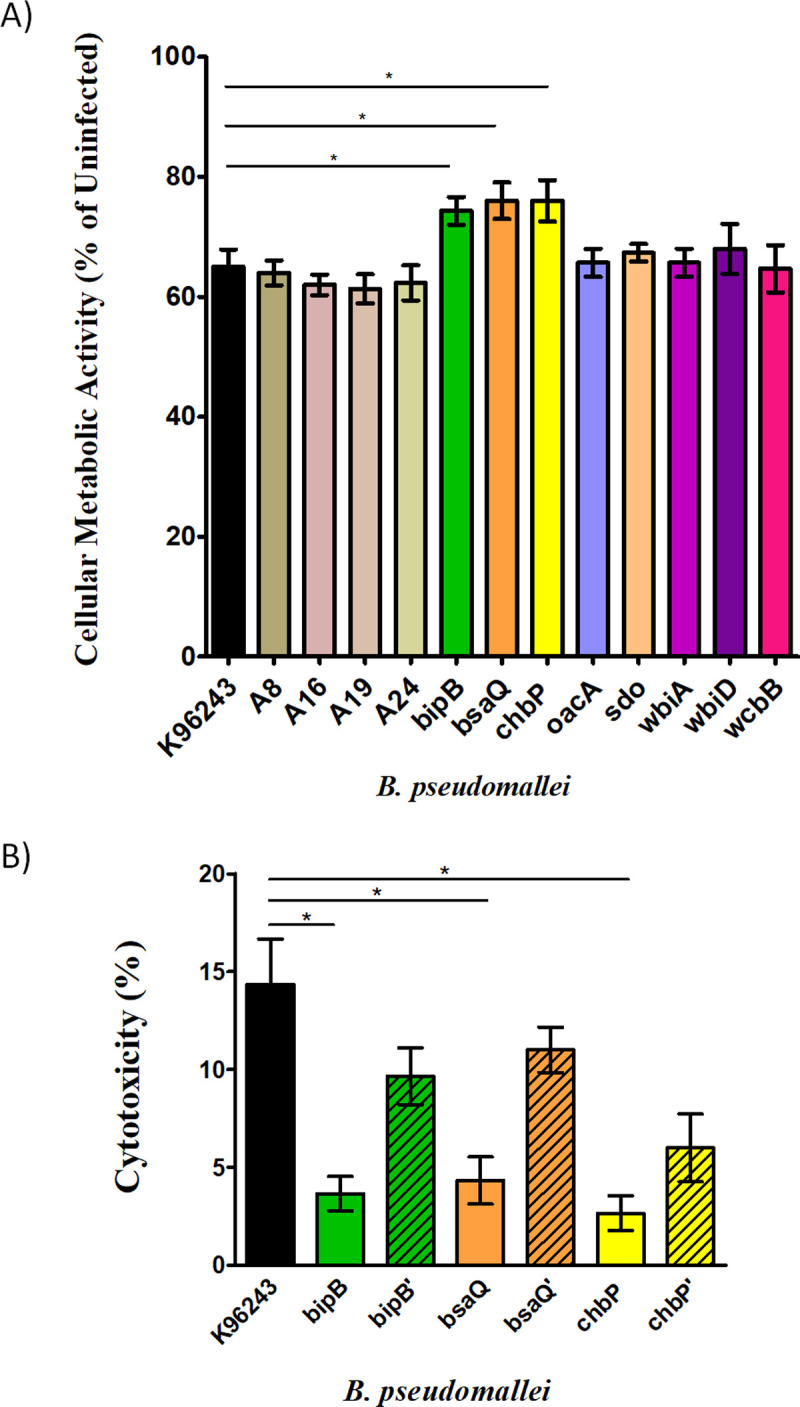
Damage of *B*. *pseudomallei*-infected fibroblasts. A) Cellular metabolic activity of *B. pseudomallei*-infected fibroblasts. HFF-1 human skin fibroblast cells were infected with reference strain K96243, *fhaB3*+ isolates (A16 and A19), *fhaB3*- isolates (A8 and A24), and eight mutant strains of *B. pseudomallei* at a MOIhttps://www.sciencedirect.com/topics/immunology-and-microbiology/multiplicity-of-infection of 20 for 10 h post-infection. The cellular metabolic activity of infected cells was measured using an MTT assay. Values represent the mean ± standard deviation from three independent experiments. * *p* ≤ 0.05 indicates a significant difference compared with reference strain K96243. B) Cytotoxicity of human skin fibroblast cells infected with *B. pseudomallei* wild-type strain K96243, Bsa T3SS mutants, and complemented strains. HFF-1 human skin fibroblast cells infected with *B. pseudomallei* K96243 and Bsa T3SS mutant strains at a MOI of 20 were used. The spontaneous release is the amount of LDH release from the cytoplasm of uninfected cells, whereas the maximum release is the amount released by total lysis of uninfected cells by Triton X-100. Values represent the mean ± standard deviation from three independent experiments. * *p* ≤ 0.05 indicates a significant difference compared with reference strain K96243.

Upon infection, damage of human skin fibroblasts may occur by successful intracellular survival of *B. pseudomallei*. Metabolism is a fundamental cellular process, reflecting that cells are still alive or damaged [32]. Cellular metabolic activity was determined in *B. pseudomallei*-infected HFF-1 human skin fibroblast cells by testing the activity of tetrazolium salt 3- (4,5-dimethylthiazol-2-yl)-2,5-diphenyltetrazolium bromide (MTT assay). The absorption value obtained with the control (uninfected) cells was adjusted to 100% cellular metabolic activity. During 4–8 h post infection, the cellular metabolic activity of *B. pseudomallei*-infected human skin fibroblast cells decreased over time, suggesting that cells were damaged (S3 Fig). However, there were no significant differences in cellular metabolism between the K96243 wild-type and other strains (S3 Fig). At 10 h, significant differences in cellular metabolism were observed between reference strain K96243 and the Bsa T3SS mutants (*p* ≤ 0.01, Fig 4A). Cellular metabolic activity was highest in human fibroblasts infected by *bipB* (74%), *bsaQ* (76%), and *chbP* (76%) mutants, which were considered to exert low cytotoxicity. However, K96243, SDO (*sdo*), OPS (*oacA*, *wbiA*, *wbiD*), and CPS (wcbB) mutants induced a significant reduction in cellular metabolic activity (64%–68%).

Since, it was clear that the Bsa T3SS mutants had a significant effect on the cellular metabolic activity of human skin fibroblasts (Fig 4A), we next investigated the integrity of the cell membrane as an indicator of cellular cytotoxicity by measuring the level of LDH released into the cellular supernatant. As expected, the percentage of cytotoxicity at 10 h post-infection by strain K96243 was significantly higher than those by the Bsa T3SS mutants (*bipB*, *bsaQ*, and *chbP*) (*p* < 0.001, 0.005, and 0.009, respectively; Fig 4B).

### MMP production by human skin fibroblasts infected by *B*. *pseudomallei*

**Fig 5:**
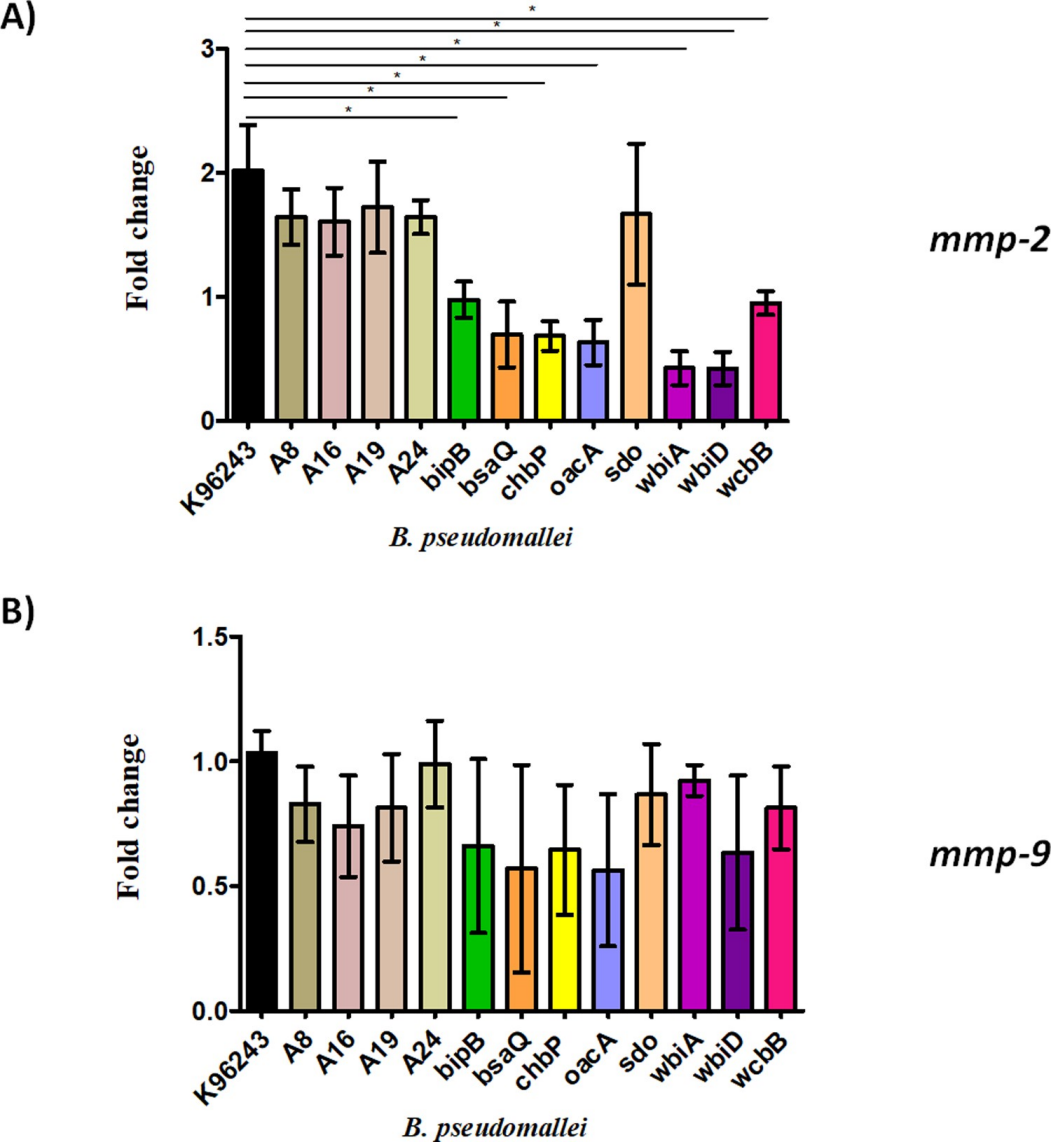
MMP gene expression by HFF-1 human skin fibroblasts infected with *B*. *pseudomallei* strains at 10 h post-infection. A) The fold change in gene expression of *mmp-2*. B) The fold change in gene expression of *mmp-9*. RPL19 was used as a reference in the calculation of relative expression levels. The normalized expression levels were calculated using the 2^˗ΔΔCT^ method. Data represent the mean ± standard deviation. * indicates statistical significance at p ≤ 0.05.

Matrix metalloproteinases (MMPs) are members of a large family of enzymes that are generally induced during cell injury and inflammatory conditions. Breakdown of cell membrane integrity has been reported to occur following the upregulation of host MMP expression in response to infection with Burkholderia spp. [33]. Especially, the gelatinases MMP-2 and MMP-9, which breakdown the extracellular matrix (ECM) components, have been displayed to be upregulated in vitro following infection with Burkholderia cenocepacia [33]. To investigate the expression of MMPs in the HFF-1 human skin fibroblasts during *B. pseudomallei* infection, the expression of MMP-2 (gelatinase B) and MMP-9 (gelatinase A) were analyzed by a real-time PCR assay. The result showed that there was no difference of MMP-2 expression observed in the infected cells by all clinical strains, both *fhaB3*+ and *fhaB3*- isolates, compared to those by the reference strains (Fig 5A). Interestingly, the expression of MMP-2 was reduced in response to infections with Bsa mutants (*bipB*, *bsaQ*, *chbP*), OPS (*oacA*, *wbiA*, *wbiD*), and CPS (wcbB) mutants. However, MMP-2 expression following SDO mutant infection showed the same level as detected in the *B. pseudomallei* K96243-infected cells (Fig 5A). By contrast, there was no significant difference in MMP-9 expression in human skin fibroblasts in response to any of the *B. pseudomallei* strains (Fig 5B).

## Discussion

*B. pseudomallei* commonly enters the body via three routes: inhalation during respiration, cutaneous inoculation or skin abrasion, and ingestion. Skin melioidosis is a rare clinical manifestation of *B. pseudomallei* infection that can be fatal to the host [11, 12, 34]; however, the underlying mechanism of *B. pseudomallei* pathogenesis during skin fibroblast infection remained poorly understood. In this study, we investigated *B. pseudomallei* pathogenesis in HFF-1 human skin fibroblasts. Previously, we have shown that *B. pseudomallei* K96243 adheres to HFF-1 cells using a MOI of 100 [18]. This study first describes the invasion efficiency of *B. pseudomallei* K963243 into the human skin fibroblast. We were able to detect the intracellular bacteria when using a MOI of 20:1 and the invasion efficiency increased using a MOI of 100 (Table 2). We cannot preclude that the invasion of human skin fibroblasts by *B. pseudomallei* can be saturated as observed during *B. cepacia* entry into epithelial cells [35].

Previous reports demonstrated the role of filamentous hemagglutinin (FhaB) in *B. pseudomallei* virulence [36, 37]. FhaB3 is an anti-macrophage factor that is encoded by BPSS2053 in *B. pseudomallei* K96243 [38]. It has been described to be involved in the attachment of host cells [37]. We investigated the role of FhaB3 in HFF-1 skin fibroblast infection using *B. pseudomallei* isolated from melioidosis patients who acquired the infection through skin abrasions. We compared the ability of *B. pseudomallei* clinical isolates possessing FhaB3 (*fhaB3*+) and lacking FhaB3 (*fhaB3*-) with the reference strain K96243 that contained FhaB3. On the basis of co-culture assays between different *B. pseudomallei* strains and HFF-1 human skin fibroblasts, we found that all *B. pseudomallei* wild-type strains tested in this study possessed the ability to invade and replicate intracellularly. These results are consistent with previous data that showed that *B. pseudomallei* E8 (FhaB3+) can invade and exhibit prolonged (≥17 h) intracellular survival in gingiva fibroblasts [29]. However, we were unable to determine this phenotype later than 10 h post-infection because MNGC formation had occurred by this time point, along with the destruction of infected cells. Interestingly, MNGC formation was not detected in infected gingiva fibroblasts in the previous study [29], whereas in our study, approximately 0.4% and 22% of MNGC formation were observed in the wild-type strains at 8 h and 10 post-infection, respectively. These findings implied that the induction of MNGC formation by *B. pseudomallei* depends on the source of host cells. In addition to invasion, replication, and MNGC formation, the cytotoxicity induced by the clinical isolates was indistinguishable from the reference strain K96243. Collectively, it is likely that FhaB3 does not affect *B. pseudomallei* invasion, replication, and MNGC formation during skin fibroblast infection. However, FhaB3 may still have a role in pathogenesis by either affecting the host immune response, or through other mechanisms not addressed in this study.

*B. pseudomallei* possesses several virulence factors that facilitate the establishment of successful infection. Here, we focused on known virulence factors including the Bsa T3SS, OPS, CPS, and SDO metabolism. We used eight *B. pseudomallei* mutants defective in the Bsa T3SS (*bipB*, *bsaQ*, *chbP*), OPS (*oacA, wbiA, wbiD*), CPS (*wcbB*), and SDO metabolism (*sdo*) to investigate the mechanism by which *B. pseudomallei* invades and survives in human skin fibroblast cells. In this study, we provide the first evidence to suggest that the Bsa T3SS plays a role in the processes of invasion, MNGC formation, and cytotoxicity of *B. pseudomallei* in human skin fibroblasts. Indeed, complementation of the *bipB*, *bsaQ*, and *chbP* mutants fully restored the induction of invasion and MNGC formation (Figs 1B, 3C, and Table 3). It also partially restored the percentage of HFF-1 cytotoxicity to wild-type levels (Fig 4B).

We showed that *B. pseudomallei* Bsa mutants had a reduced ability to invade human skin fibroblasts, similar to the role of the Bsa T3SS in the pathogenesis of *B. pseudomallei* in HeLa cells, A549 cells, and J774.1 macrophages [9, 16, 39]. Previously, it was shown that the *bopE* mutant, which is defective in producing the BopE effector of the Bsa T3SS, had a reduced capacity for bacterial invasion into HeLa cells [10]. Similarly, a *B. pseudomallei bsaQ* mutant, which is unable to produce the structural component of the Bsa T3SS, demonstrated decreased plaque formation and invasion into non-phagocytic cells [14]. By contrast, the *Salmonella* T3SS expressed by pathogenicity island 1 did not appear to play an essential role in bacterial invasion into fibroblasts that were obtained from different sources [40]. This phenomenon indicates that different bacteria might employ different mechanisms for invading host cells.

*B. pseudomallei* can multiply and survive inside phagocytic and non-phagocytic cells [8, 26]. According to our findings in an intracellular replication assay, *B. pseudomallei* can proliferate and survive inside the HFF-1 human skin fibroblasts. Upon contact with host cells, the Bsa T3SS is activated and effector proteins of *B. pseudomallei* are secreted that aid survival inside host cells [41]. *B. pseudomallei* Bsa T3SS mutants (*bipB*, *bsaQ*, and *chbP*) were not defective in their ability for intracellular replication in HFF-1 skin fibroblasts (Fig 2B and S2 Table). In accordance with a previous study, *B. pseudomallei bsaQ*, shown to have reduced invasion, was able to survive and replicate inside A549 epithelial cells and J774A.1 macrophages [14].

Furthermore, *B. pseudomallei* Bsa T3SS was required for *B. pseudomallei* to escape from endocytic vesicles and survive in the cytoplasm of J774.2 murine macrophage‐like cells [42], as well as MNGC formation of J774A.1 macrophages [15]. Interestingly, we found that *B. pseudomallei* Bsa T3SS mutants impaired the MNGC formation in HFF-1 skin fibroblasts. The role of Bsa T3SS in inducing MNGC formation was consistent with a previous study that showed that mutation of *bipB* and *bsaQ* resulted in decreased MNGC formation in J774A.1 cells [15, 14]. Bsa T3SS mutants also showed a reduced capacity to induce MNGC formation in HFF-1 skin fibroblasts.

*B. pseudomallei* also increased LDH release and decreased cellular metabolic activity during HFF-1 skin fibroblast infection. This indicates that *B. pseudomallei* can invade, replicate, and induce the formation of MNGC, eventually leading to host cytotoxicity and cell damage. Although the role of cell damage in *B. pseudomallei* pathogenesis is uncertain, the disruption of skin fibroblast membranes might enhance inflammation and the release of enzymes contributing to local tissue destruction. Furthermore, lysis of skin fibroblasts might also facilitate bacterial entry into the bloodstream, leading to systemic spread.

Indeed, we found that the HFF-1 human skin fibroblasts infected with *B. pseudomallei* lacking *bipB*, *bsaQ*, and *chbP*, showed reduced LDH production. For that reason, we propose that the Bsa T3SS of *B. pseudomallei* causes damage to human skin fibroblasts and that the damage of cells in response to infection is associated with the induction of the MMP enzymes. These enzymes digest collagen and other ECM components, which are important for a wide range of processes, including cell proliferation, migration, apoptosis, regeneration, and inflammation [43]. During bacterial infection, MMPs induce cellular changes during inflammation that facilitate bacterial invasion [33]. The damage of skin fibroblasts during infection can support bacterial spread into the circulatory system, thereby contributing to the severity of the disease. In this study, we investigated the expression of MMPs after the infection of human skin fibroblasts by *B. pseudomallei*. The *mmp-2* gene was shown to be induced in HFF-1 human skin fibroblast cells infected with *B. pseudomallei* strains. In contrast, the *mmp-9* gene was not upregulated in the present study. Previously, there was a report of upregulation of MMP-2 and MMP-9 during the infection of host cells with *B. cenocepacia* [33]. This implied that the expression pattern of MMPs is diverge among cell types, as the MMPs in HFF-1 skin fibroblast response do not respond in the same fashion, but rather are differentially regulated. In addition, *B. pseudomallei* defective in the Bsa T3SS, OPS, and CPS demonstrated reduced expression of the *mmp-2* gene. Taken together, it is possible that these virulence factors may be linked to the damage of skin fibroblast following the infection of *B. pseudomallei*. Nevertheless, other undefined virulence factors may be important for *B. pseudomallei* infection of skin fibroblasts.

OPS, CPS, and SDO are well-characterized virulence factors that are important for *B. pseudomallei* infection [19, 44]. For example, one study demonstrated that CPS plays a role in *B. pseudomallei* survival during respiratory infection [44]. OPS plays a significant role in antigenic variation and adherence of *B. pseudomallei* [17]. The importance of SDO was linked with the phenotype of SDO mutant, which showed the defective abilities in the adhesion to HFF-1 human skin fibroblast cells [18], invasion into A549 lung epithelial cells [19], and early intracellular survival within J774A.1 macrophage cells [19]. However, our findings indicated that OPS, CPS, and SDO were not likely involved in the invasion, intracellular survival and MNGC formation of *B. pseudomallei* during HFF-1 skin fibroblast infection. OPS and CPS might be associated with *B. pseudomallei*-induced MMP production in skin fibroblasts. Further studies are required to elucidate the role of OPS and CPS in MMP expression.

In conclusion, we demonstrated that HFF-1 human skin fibroblast cells are susceptible to several strains of *B. pseudomallei*, which can invade, replicate, induce the formation of MNGC, and cause cellular damage. The Bsa T3SS is potentially a virulence factor of *B. pseudomallei* during the process of skin fibroblast infection. We also found that this host cell type responds to the bacteria by expressing MMP. Our findings provide a better understanding of *B. pseudomallei* pathogenesis in skin fibroblasts, which may have important implications in preventing the progress of cutaneous melioidosis.

## Supporting information

S1 Fig Growth kinetics of *B*. *pseudomallei* strains. Thirteen *B. pseudomallei* strains were cultured in LB broth and RPMI medium at 37°C with shaking. OD was determined at 600 nm.
(TIF)

S2 Fig Transmission electron micrograph of intracellular *B*. *pseudomallei* within human skin fibroblast cells. HFF-1 human skin fibroblasts were infected with *B. pseudomallei* K96243 at a MOI of 20. This representative image of internalized *B. pseudomallei* was taken at 4 h post-infection under a H7700 transmission electron microscope at a 2000× magnification.
(TIF)

S3 Fig Cellular metabolic activity of *B*. *pseudomallei*-infected fibroblasts at different time points. HFF-1 human skin fibroblast cells were infected with reference strain K96243, *fhaB3*+ isolates (A16 and A19), *fhaB3*- isolates (A8 and A24), and eight mutant strains of *B. pseudomallei* at a MOI of 20 for 4, 6, and 8 h post-infection. The cellular metabolic activity of infected cells was measured using an MTT assay. Values represent the mean ± standard deviation from three independent experiments. * *p* ≤ 0.05 indicates a significant difference compared with reference strain K96243.
(TIF)

S1 Table Oligonucleotide primers used in this study [46]. (DOCX)

S2 Table Intracellular *B*. *pseudomallei* was enumerated in HFF-1 human skin fibroblasts during 10 h post-infection, generation time, and fold change in cfu was determined compared to 4 h post-infection. (DOCX)
