# Supplementary figures and images for: Burkholderia pseudomallei pathogenesis in human skin fibroblasts: A Bsa type III secretion system is involved in the invasion, multinucleated giant cell formation, and cellular damage
Source: PLoS One. 2022 Feb 3;17(2):e0261961. doi: 10.1371/journal.pone.0261961 (PMC8812868; doi:10.1371/journal.pone.0261961)

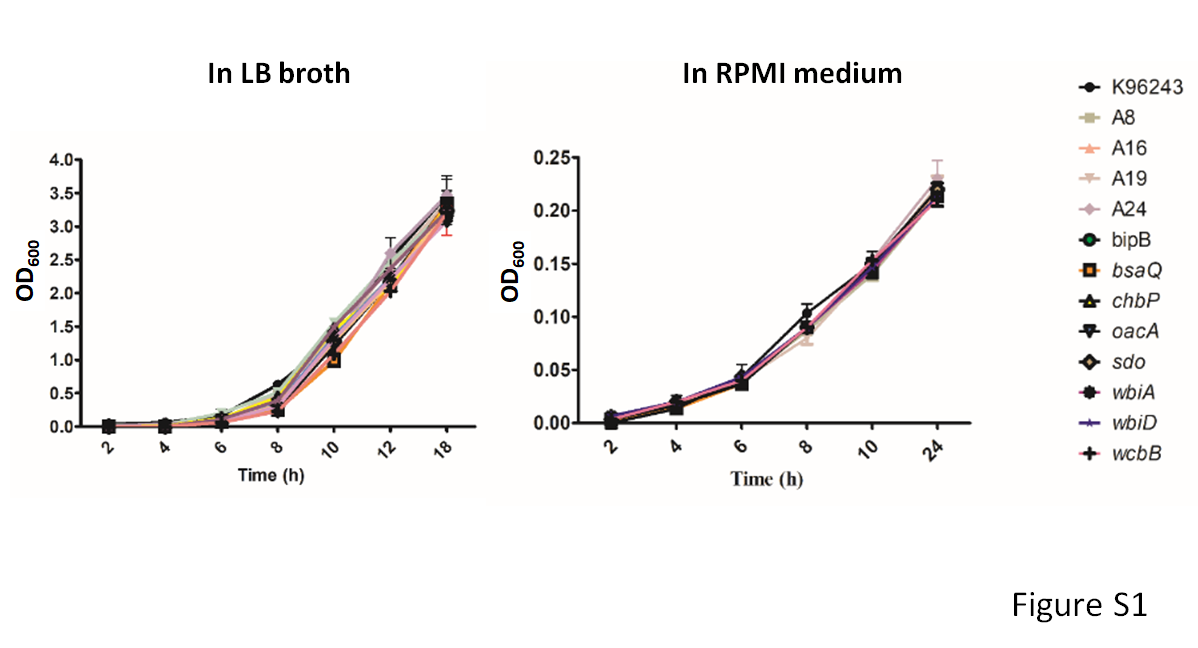

Supplement: S1 Fig — Thirteen B. pseudomallei strains were cultured in LB broth and RPMI medium at 37°C with shaking. OD was determined at 600 nm. (TIF) [file pone.0261961.s001.tif]

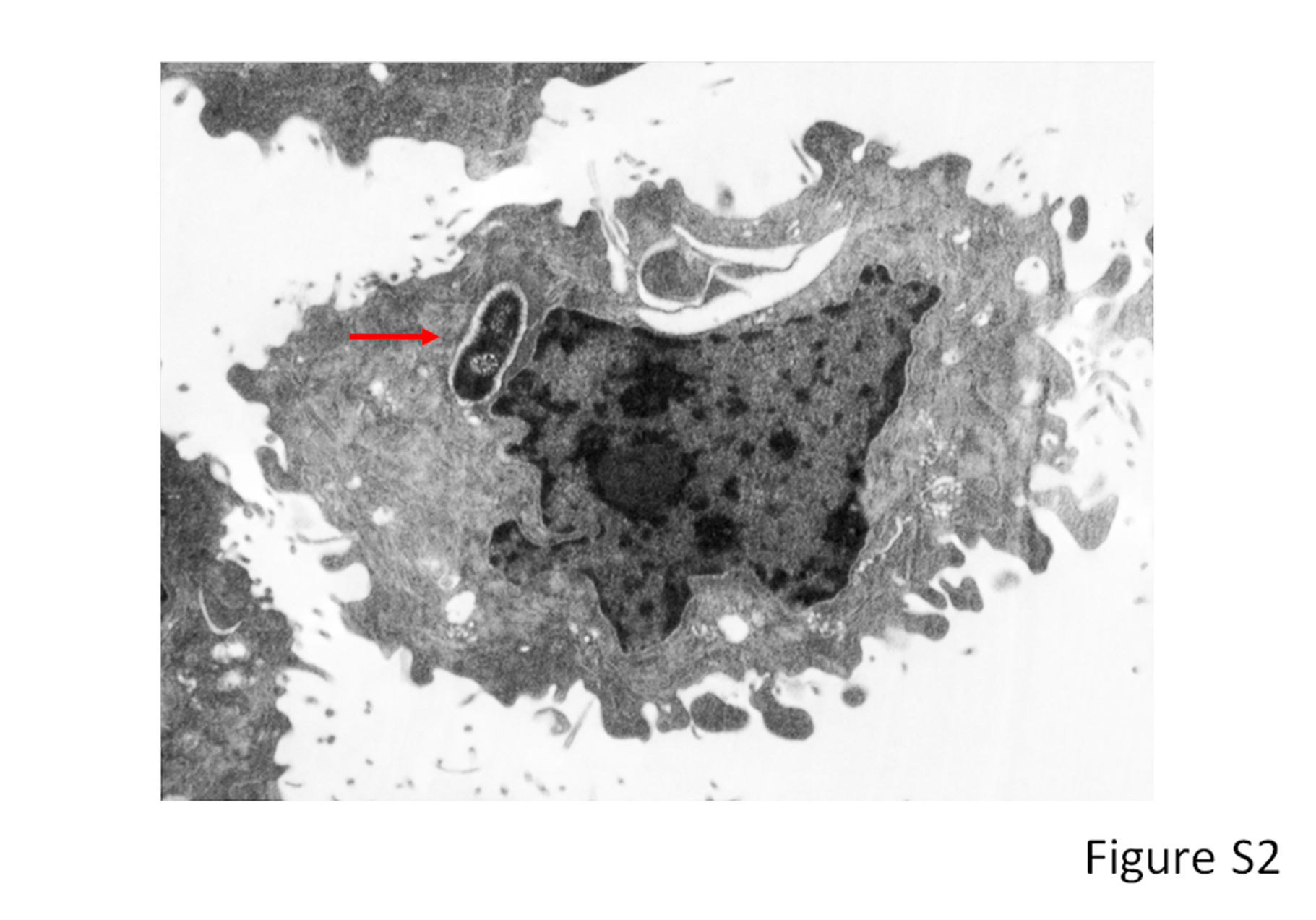

Supplement: S2 Fig — HFF-1 human skin fibroblasts were infected with B. pseudomallei K96243 at a MOI of 20. This representative image of internalized B. pseudomallei was taken at 4 h post-infection under a H7700 transmission electron microscope at a 2000× magnification. (TIF) [file pone.0261961.s002.tif]

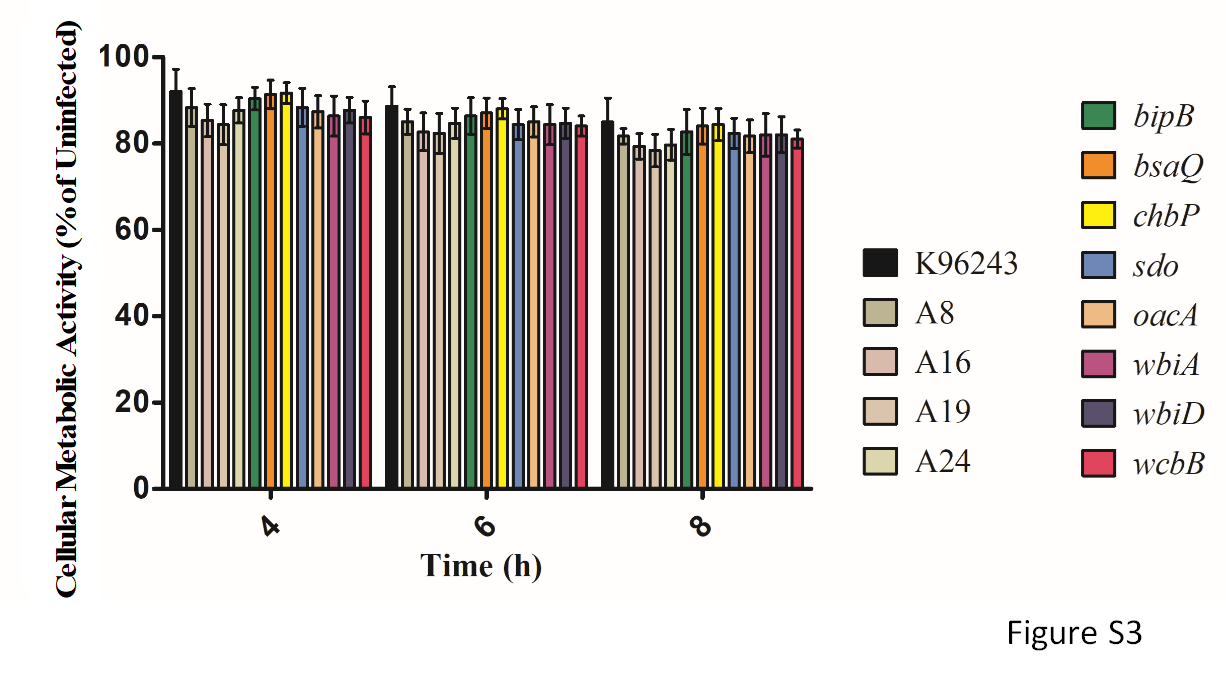

Supplement: S3 Fig — HFF-1 human skin fibroblast cells were infected with reference strain K96243, fhaB3+ isolates (A16 and A19), fhaB3- isolates (A8 and A24), and eight mutant strains of B. pseudomallei at a MOI of 20 for 4, 6, and 8 h post-infection. The cellular metabolic activity of infected cells was measured using an MTT assay. Values represent the mean ± standard deviation from three independent experiments. * p ≤ 0.05 indicates a significant difference compared with reference strain K96243. (TIF) [file pone.0261961.s003.tif]
