# Supplementary material for: Burkholderia pseudomallei pathogenesis in human skin fibroblasts: A Bsa type III secretion system is involved in the invasion, multinucleated giant cell formation, and cellular damage
Source: PLoS One. 2022 Feb 3;17(2):e0261961. doi: 10.1371/journal.pone.0261961 (PMC8812868; doi:10.1371/journal.pone.0261961)
Supplement: S1 Table — (DOCX) [file pone.0261961.s004.docx]

**S1 Table. Oligonucleotide primers used in this study.**

| **Gene** | **Primers sequences (5’–3’)** | **Sources** |
| --- | --- | --- |
| *bipB* | F-BipB-BamHI: cgtggatcCCCGTATTACACTGCGCGGG | This study |
|  | R-BipB-EcoRI:  gagctgaattcATGTCATCGGGAGTGCAGG |  |
| *bsaQ* | F-BsaQ-BamHI: catcggatccCTCCTTAGATCGTCTTCAACACA |  |
|  | R-BsaQ-SmaI: ggcccgggGTCATGCTGAAGAATCTCCTGA |  |
| *mmp-2* | F: ATAACCTGGATGCCGTCGT  R: AGGCACCCTTGAAGAAGTAGC | [46] |
| *mmp-9* | F: GAACCAATCTCACCGACAGG  R: GCCACCCGAGTGTAACCATA |  |
| *RPL19* | F: GTGGCAAGAAGAAGGTCTGG  R: GCCCATCTTTGATGAGCTTC |  |
| MMP, matrix metalloproteinase; RPL19, ribosomal protein L19;  F, forward; R, reverse. | | |
