# Supplementary material for: Burkholderia pseudomallei pathogenesis in human skin fibroblasts: A Bsa type III secretion system is involved in the invasion, multinucleated giant cell formation, and cellular damage
Source: PLoS One. 2022 Feb 3;17(2):e0261961. doi: 10.1371/journal.pone.0261961 (PMC8812868; doi:10.1371/journal.pone.0261961)
Supplement: S2 Table — (DOCX) [file pone.0261961.s005.docx]

**S2 Table.** **Intracellular *B. pseudomallei* was enumerated in HFF-1 human skin fibroblasts** **during 10 h post-infection, generation time, and fold change in cfu was determined compared to 4 h post-infection.**

| ***B. pseudomallei* strains** | **Average number of**  **intracellular bacteria (cfu)** | | | | **Doubling time**  **(min)** | **Fold increase**  **(compared to 4 h)** | | |
| --- | --- | --- | --- | --- | --- | --- | --- | --- |
|  | **4 h** | **6 h** | **8 h** | **10 h** |  | **6 h** | **8 h** | **10 h** |
| **K96243** | 2933 | 5633 | 68333 | 693333 | 64.48 | 1.9205 | 23.2955 | 236.364 |
| **A8** | 1900 | 3900 | 53000 | 536666 | 63.81 | 2.0526 | 27.8947 | 282.456 |
| **A16** | 2833 | 5166 | 60333 | 580000 | 65.19 | 1.8235 | 21.2941 | 204.706 |
| **A19** | 1766 | 3733 | 49000 | 496666 | 63.98 | 2.1132 | 27.7358 | 281.132 |
| **A24** | 2300 | 3816 | 51666 | 503333 | 64.15 | 1.6594 | 22.4638 | 218.841 |
| ***bipB*** | 1766 | 1933 | 27000 | 263333 | 63.81 | 1.0943 | 15.2830 | 149.057 |
| ***bsaQ*** | 1666 | 1800 | 20666 | 243333 | 63.98 | 1.0800 | 12.4000 | 146.000 |
| ***chbP*** | 1200 | 1200 | 6633 | 108666 | 66.87 | 1.0000 | 5.5278 | 90.556 |
